# Comparative Analysis of the Liquid CO_2_ Washing with Conventional Wash on Firefighters’ Personal Protective Equipment (PPE)

**DOI:** 10.3390/textiles2040036

**Published:** 2022-11-25

**Authors:** Arjunsing Girase, Donald Thompson, Robert Bryan Ormond

**Affiliations:** Textile Protection and Comfort Center, Wilson College of Textiles, North Carolina State University, Raleigh, NC 27695, USA

**Keywords:** fireground contaminants, liquid CO_2_, NFPA 1851, carcinogenic, cleaning efficiency, PAHs, phenols, phthalates

## Abstract

Firefighters are exposed to several potentially carcinogenic fireground contaminants. The current NFPA 1851 washing procedures are less effective in cleaning due to the limited intensity of the washing conditions that are used. The 2020 edition of NFPA 1851 has added limited specialized cleaning for higher efficacy. The liquid carbon dioxide (CO_2_) laundering technique has gained popularity in recent years due to its availability to remove contaminants and its eco-friendliness. The primary aim of this study is to address the firefighter questions regarding the efficacy of cleaning with liquid CO_2_ and to compare it with the conventional washing technique. The unused turnout jackets were contaminated with a mixture of fireground contaminants. These turnout jackets were cleaned with conventional NFPA 1851-appoved aqueous washing and a commercially available liquid CO_2_ method. Post-cleaning samples were analyzed for contamination using pressurized solvent extraction and GC-MS. The liquid CO_2_ technique demonstrated considerable improvement in washing efficiency compared to the conventional washing.

## 1. Introduction

The International Agency for Research on Cancer (IARC) has stated that the profession of firefighting is a known carcinogen to human beings [1]. Firefighters are exposed to several chemicals during fire suppression activities. Polycyclic aromatic hydrocarbons (PAHs) are compounds that are released due to the incomplete combustion of materials. PAHs have toxic and mutagenic properties while some of them are endocrine disruptors. Multiple PAHs, including the known carcinogen benzo[a]pyrene, have been found in firefighters’ personal protective equipment (PPE) and on their skin [2]. Among several different compounds, plasticizers are also found on firefighter PPE. When used samples of PPE were analyzed, 20 different PAHs and 6 phthalate esters were found. Phthalates are ubiquitously found in polyvinyl plastic materials that are used abundantly in flooring, wire sheathing, and home furnishings [3].

The NFPA 1851 Standard on Selection, Care, and Maintenance of Protective Ensembles for Structural Fire Fighting and Proximity Fire Fighting has standard guidelines and requirements for inspecting, cleaning, and maintaining firefighter turnout gear. These guidelines include washing at temperatures less than 105 °F (40 °C), G-forces in the extractor should be less than 100 G, and prohibiting the use of bleaching or oxidizing agents [4]. The standard has categorized the decontamination techniques as (1) Preliminary Exposure Reduction (PER), (2) Advanced Cleaning, (3) Specialized Cleaning. The advanced cleaning procedure permits the use of programmable washing machines and detergents. The specialized cleaning is performed by a verified independent service provider (ISP). The standard clearly states to use specialized cleaning when the ensemble is inadequately cleaned by advanced cleaning [4].

A limited number of studies have been conducted that indicate residual contamination after using the standard aqueous wash. Mayer et al., 2019 investigated the impact of routine laundering on firefighter hoods. The study was performed on two sets of hoods that were exposed to the same structural fire. One set was routinely laundered after every fire scenario and in total, was washed four times in a standard washer extractor. The other set was kept unlaundered to assess the contamination. The analysis between the two sets showed that overall, laundered hoods had 81% lower PAH contamination than unlaundered hoods. The pre-wash and post-wash analyses were performed on completely different sets of hoods. The high values of standard deviation in contamination indicated high spatial variability that may have affected washing efficiency results [5]. A study of water-only decontamination of turnout gear used in live-structure burns showed an increase in contamination by 42%; however, this increase could have been attributed to the disparity in sampling sites for pre- and post-washing samples [6]. Thus, the uneven contamination on the gear is a major hindrance in calculating the washing efficiency of the method and prevents from gaining a comprehensive understanding of the process. The above studies indicated a need for a controlled assessment that includes uniform contamination and targeted contaminants as opposed to using the highly variable live-fire scenarios to contaminate the materials.

Dry cleaning is a technique of removing soils and contaminants from textiles using a non-aqueous solvent. In conventional dry cleaning, perchloroethylene (PER) is most commonly used. PER has a toxic effect on the human body. Several alternatives have been looked at for textile dry cleaning applications such as hydrocarbon solvents, Green earth^®^, acetal silicon-based solvents, and carbon dioxide (CO_2_) [7]. CO_2_ has distinctive advantages over other solvents such as being non-toxic, non-flammable, non-corrosive, environmentally benign, and economical [8]. Some studies have indicated that the cleaning efficiency of CO_2_ for non-particulate soil removal is comparable to that of preliminary exposure reduction, which is conducted on-scene with a brush, soap, and water. The particulate removal for CO_2_ dry cleaning was lower [8]. For dry cleaning operations, the liquid state of CO_2_ is preferred over the supercritical state since the two-phase gas-liquid interface is beneficial for trapping soil particles. The substantially higher pressure in CO_2_ cleaning makes it easy to separate the CO_2_ from the detergent formulation and the soil post-cleaning process. Additionally, the spontaneous evaporation of CO_2_ from the fabric during depressurization saves the energy of drying [9].

The following study was conducted to evaluate and compare the cleaning efficacies of liquid CO_2_ washing and conventional aqueous wash for the application of firefighter protective clothing. The novelty of the experiment is that controlled contamination of the targeted fireground contaminants is used to evaluate two different washing techniques: Conventional wash and liquid CO_2_, which is a novel technique to clean the fireground contaminants and has not been used and compared with conventional wash. The null hypothesis for the experiment was that there is no significant difference in cleaning efficacies of conventional and liquid CO_2_ methods.

## 2. Materials and Methods

**Figure 1.**
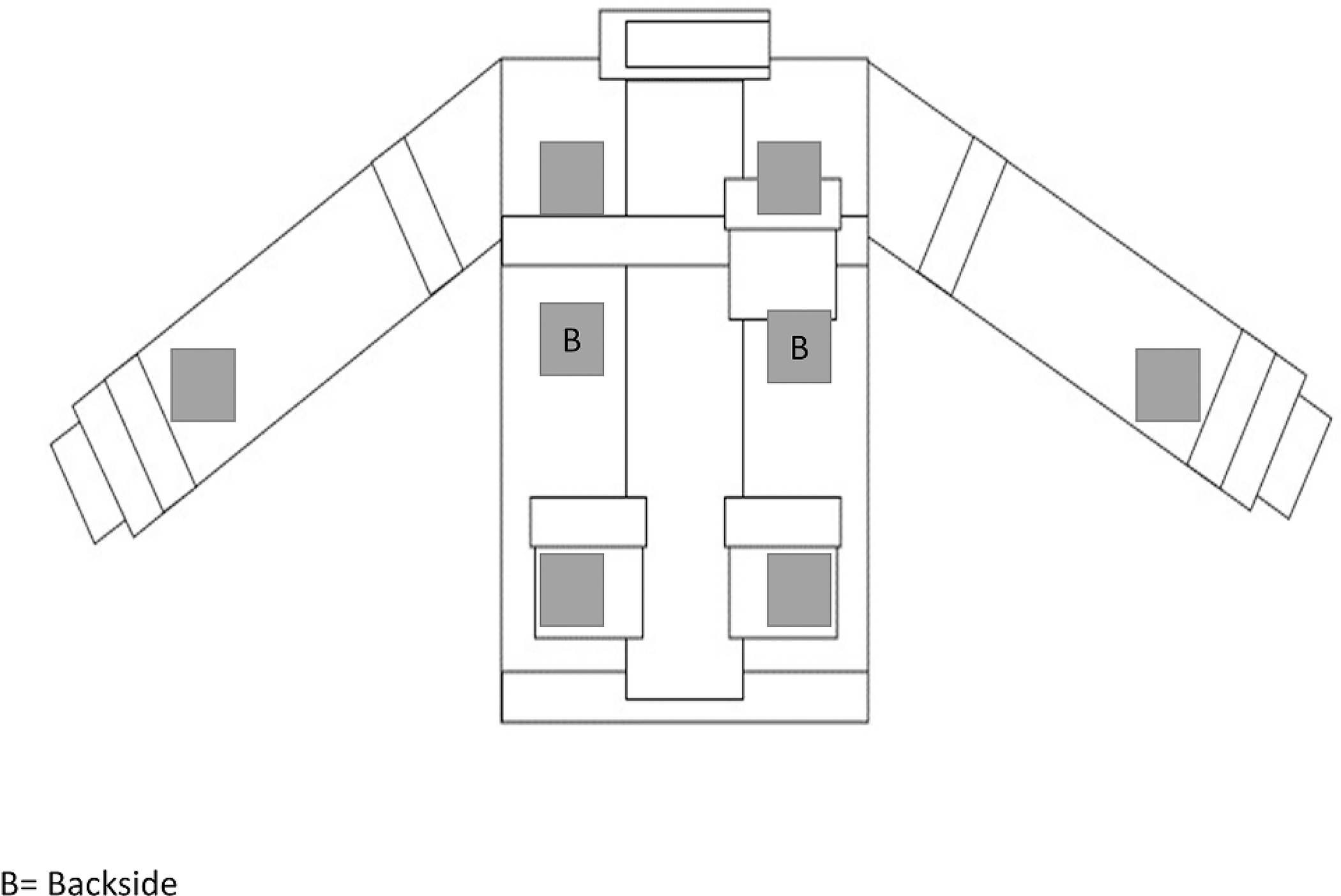
Schematic of the turnout suit.

For this study, five new turnout jackets were used to mount the test samples (swatches) for cleaning. On every single jacket, eight hook-and-loop patches were stitched. The hook part was stitched on the jacket and the loop part was stitched to the test samples. The positions of the hook-patches are shown in the schematic in Figure 1. The size of each patch was 5 cm × 5 cm. The test sample swatches (5 cm × 5 cm) were prepared separately using the outer shell material, PBI Max^™^ Gold (6 oz.), with a fluorinated durable water-repellent finish. These swatches were spiked with targeted fireground contaminants using analytical standards (Table 1). Three analytical standards for phenols, PAHs, and phthalates (2000 ng/μL for each compound in the mix) were used to contaminate the test samples. The solutions were diluted to 1000 ng/μL using n-hexane. Twenty 3-μL drops of each standard mix were applied on the swatch from the stock solution using a repeater pipette. Thus, the amount of each contaminant present on a single test sample was 60,000 ng. All the test samples were allowed to dry for 24 h. After contamination, the test samples were adhered to the positions on the turnout jackets. In addition to the contaminated swatches, blank samples were shipped in a plastic bag along with the samples for liquid CO_2_ cleaning to identify any cross-contamination during transportation. Similarly, positive control (contaminated-but-not-washed) fabric samples were prepared before the extraction process.

### 2.1. Liquid CO_2_ Protocol

To conduct the liquid CO_2_ cleaning of the test samples, the research team employed Tersus Solutions (Denver, CO, USA). All of the test jackets were shipped to the cleaning facility to be washed with liquid CO_2_ utilizing a protocol that is proprietary to the facility. The limited details of the method are given in Table 2. After washing, all the test samples attached to the jackets were sent back for analysis. These test samples were removed from the jackets and stored separately in the plastic bag and in the refrigerator at 4 °C for 24 h after receiving. The analysis was done using the analytical method described later in the article.

### 2.2. Conventional Washing Protocol

For comparative analysis, the test sample preparation process was repeated exactly for the samples to receive conventional aqueous wash using a commercially available detergent (CD-1). The ingredients of CD-1 are shown in Table 3. The UNIMAC^®^ 45 lbs. washing extractor was used in this process. The machine was installed in Wilson College of Textiles, Raleigh, NC. The temperature of the wash was kept at 40 °C (105 °F) and the duration of the wash was 60 min. The conventional method was compliant with the NFPA 1851 requirements. Due to the limited availability of the materials (liquid contaminants, unused jackets, velcro patches), the jacket was patched with five contaminated swatches. The positions where test samples were attached were chosen randomly. The amount of detergent CD-1 used was 120 mL per 45 lbs. load and was calculated according to the manufacturer’s recommendations. All test samples were air-dried after washing for 24 h and then extracted and analyzed as described below.

### 2.3. Extraction

All fabric samples were extracted using a pressurized solvent extractor (BUCHI^®^, Speed Extractor E-916) with n-hexane (Fisher Scientific, Hampton, NH, USA) as the extraction solvent. Outer shell fabrics (pre-wash or post-wash) of size 5 cm × 5 cm were placed in the 10-mL stainless steel extraction cell. Glass beads (4 mm diameter) were sonicated with n-hexane to remove any prior contamination, and 5 g of glass beads were filled inside each cell to fill the void volume to reduce the excess solvent entering the cell. The cell was capped with top and bottom cellulose filters to prohibit unwanted particulate contamination. Each extraction comprised two full extraction cycles and one flush cycle at the end. Every single extraction cycle consisted of a five-minute heat-up followed by a five-minute hold where the solvent and fabric were in contact with each other. The cycle was held at 100 °C and 100 bar, and the extraction was carried out in the nitrogen atmosphere. The extract passed through a condensing coil and was collected in a 60-mL glass vial. The total run time for the extraction process was 32 min.

After collecting, the extract from each cycle was diluted to 10 mL in a standard 10-mL volumetric flask using n-hexane. A sample of the diluted extract was transferred into the 2-mL amber autosampler vial using a 3-mL syringe with 0.2 μm PTFE filters. These vials were loaded on to GC-MS system and analyzed. All the extraction cycles included a positive control (contaminated-but-not-washed) fabric sample and a negative control (uncontaminated and unwashed) fabric sample. The post-washing concentrations of the washed samples were calculated relative to the positive control during that particular extraction. The negative controls were used to check any compounds were present on the fabrics themselves in the first place. They were used only for qualitative analysis.

### 2.4. Gas Chromatography/Mass Spectrometry

The analysis of the fireground contaminants was carried out using an Agilent 7890B Gas Chromatograph (GC) system coupled to an Agilent 5977B Mass Spectrometer (MS) equipped with Electron Ionization (EI) capability (Agilent Technologies, Inc., Santa Clara, CA, USA ). Chromatographic analysis was conducted in the splitless mode with a purge flow of 100 mL/min at 1.0 min. The Agilent Ultra Inert liner (5190–6168, straight 2 mm ID) was used in the GC inlet, which was maintained at 250 °C. An Agilent EPA 8270D fused silica capillary column (30 m × 0.25 mm × 0.25 μm) was used with a helium flow rate of 1.2 mL/min. The oven program was set to begin at 40 °C, then increased to 280 °C at a rate of 10 °C/min with a 1 min hold, followed by a further increase to 300 °C at 25 °C/min with a final hold of 1 min. The total running time was 30.48 min. The MS transfer line was kept at 280 °C throughout the run. The MS quad temp was maintained at 300 °C, and the ion source temp was kept at 200 °C. The gain factor used was 1.00. The analysis was conducted in scan mode (35–550 amu) using EI with an energy of 70 eV. The calibration solutions were prepared to calibrate the instruments using the mix of the compounds (2000 ng/μL) as the stock solutions. The calibration standards, as shown in Table 4, were prepared by diluting n-hexane (Fisher Scientific—95% purity) in a 10-mL volumetric flask. The calibration curve was obtained by averaging out the responses of three replicates. The limit of detection (LOD) and limit of quantitation (LOQ) values were calculated using Equations (1) and (2), respectively. The lowest concentration was run ten times and the standard deviation of the response (area) σ was calculated. The calibration curve provided the equation of the line which provided the slope m.

(1) LOD=3σ/m

(2) LOQ=10σ/m

### 2.5. Data Analysis

Microsoft Excel was used to calculate the $R^{2}$, slope (m) of the calibration curve, standard deviation of the responses (σ), LOD and LOQ. The linearity of the calibration solutions (response vs. concentration) was high, as indicated from the $R^{2}$ values in Table 1 for all the compounds. This showed that the proportion of the variation in the response generated for various concentrations was predictable and dependent. To quantify the effectiveness of both decontamination methods, the washing efficiency was calculated using Equation (3). For samples that did not show detectable peaks in the chromatogram, $\frac{1}{2}$ LOQ values were used in the equation. For every compound, the arithmetic mean (average) of the washing efficiencies of the replicates was calculated. For liquid CO_2_ washed samples, the average of the washing efficiency for any compound was calculated using 40 replicates and for conventional wash, the values of washing efficiency are the average of the 5 replicates. In a separate analysis, data set from five random samples were taken from the liquid CO_2_ set and compared with conventional washed samples to perform a comparative analysis at the equal number of data points and to see if that created any difference. Standard errors were calculated to check the variability across samples of the given population. The statistical analysis was done using JMP Pro^®^ statistical software (15.2.0, SAS Institute Inc., Cary, NC, USA). Shapiro–Wilks test was used to check the normal distribution of the data. A single factor ANOVA was conducted to test the variances at p<0.05, confirming the unequal variances for the one tailed *t*-test. A singled tail *t*-test was done at alpha-level = 0.05. Random sample picking from the given data set was done using JMP Pro^®^ (15.2.0, SAS Institute Inc., Cary, NC, USA).

(3) $$Cleaning\;efficiency(\%)=\frac{\left(\text{Original}\;\text{concentration}(Cc)\;-\;\text{post}\;\text{washing}\;\text{concentration}(Cw)\right)}{\text{Original}\;\text{concentration}}\times 100$$

## 3. Results

**Figure 2.**
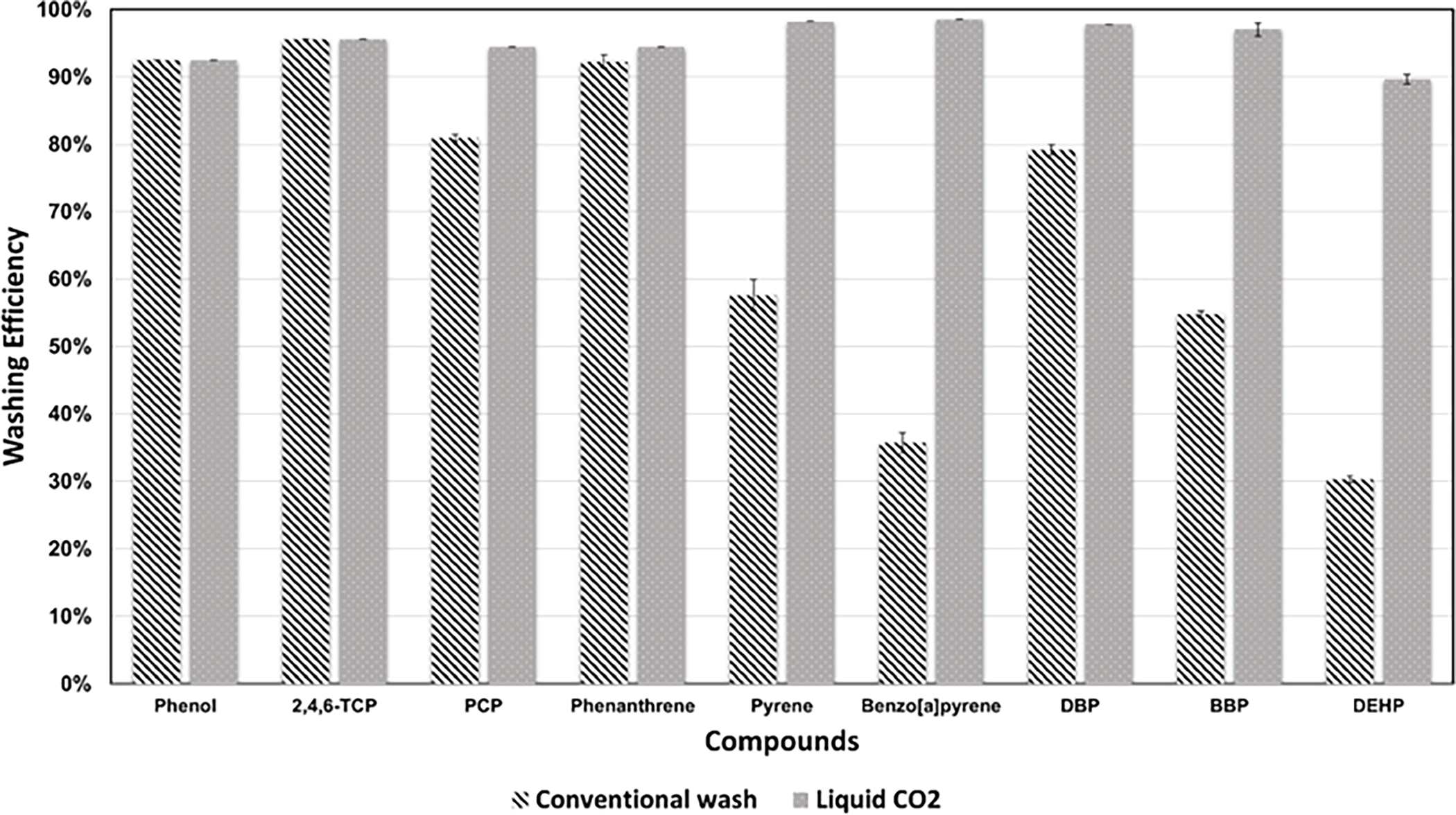
Comparison of washing efficiencies for conventional wash and liquid CO_2_.

The original target concentration applied to the materials (accounting for analytical sample preparation) for all the samples was 6 ng/μL. The blank samples in the plastic bag did not show any compounds, thus indicating no issues with cross-contamination during transportation. The washing efficiency values for both the methods: liquid CO_2_ and conventional wash are presented in Table 5 for the targeted contaminants. The comparative analysis of the washing efficiencies is shown in Figure 2 (Average washing efficiencies in Figure S1). The results for the washing efficiencies were very close together as indicated by the error percentage from Table 5 which indicates low variation. The Shapiro–Wilks test indicated a *p*-value of 0.24 > 0.05 (for Conventional wash) and a *p*-value of 0.41 > 0.5 (for liquid CO_2_ wash), indicating that the data was normally distributed (Figure S2). The single-tailed *t*-test indicated that the difference was statistically significant for p<0.05 (Figure S3). Thus, we reject our null hypothesis and conclude that there is a significant difference between the means of the different washing methods and liquid CO_2_ is more effective than the conventional washing method. For the equal number of samples where for liquid CO_2_, average of 5 random replicates was taken, similar results were found (Average washing efficiencies in Figure S4). The Shapiro–Wilks indicated *p*-value of 0.27 > 0.05 (Conventional wash) and *p*-value of 0.46 > 0.05 (liquid CO_2_ wash) (Figure S5) and the single-tailed *t*-test showed (Figure S6) that the difference between the means was statistically significant. This indicated that irrespective of the number of samples, liquid CO_2_ removed contaminants effectively.

## 4. Discussion

For conventional wash, the washing efficiency decreased from phenols to phthalates. The increase in the octanol-water partition coefficient $\left(K_{OW}\right)$ values and the decrease in the washing efficiency in a chemical class showed that the relation between the two was evident. The average washing efficiency for conventional wash is shown in Figure 2. The $\frac{1}{2}$ LOQ values from Table 1 were used for calculating phenol and TCP. The conventional wash removed these contaminants below the quantitation limits of the analytical method. The aqueous wash and non-ionic surfactants removed the phenols, phenanthrene and DBP. Phenols are more polar as compared to the other two groups. They are fairly soluble in water; hence, the results were comparable. The detergent contained d-limonene for aqueous washing, a non-polar compound that helped remove the phenanthrene and DBP. For PAHs, an increase in the number of aromatic rings increased hydrophobicity and resulted in decreased removal from the fabric. Thus, a decreasing trend in washing efficiency can be seen in the aqueous washing [10]. A similar trend was observed in phthalates, an increase in alkyl chain length increased the hydrophobicity and thus phthalates were not removed effectively by aqueous washing [11].

From the comparative analysis perspective, Figure 2 demonstrated that conventional wash was not very effective at removing higher molecular weight PAHs and phthalates. This limitation can be attributed to the polar nature of the water and the hydrophobic nature of the compounds.

For liquid CO_2_, the $\frac{1}{2}$ LOQ values were used in calculations for all the compounds except for BBP and DEHP, as they were the only compounds that had detectable levels remaining in the fabric. It indicated that the contaminants might be present in trace amounts after washing that cannot be quantified by the analytical method. Even for BBP and DEHP, the average washing efficiency was still greater than 90%.

The results indicate the potency of the liquid CO_2_ method in removing the fireground contaminants. The three different chemical groups: phenols, PAHs, and phthalates were all removed effectively using the liquid CO_2_ washing method. This may be due to the non-polar nature of liquid CO_2_ that aided in solubilizing the more hydrophobic contaminants. The proprietary detergent used has been effective in removing phenols. The high diffusivity and low viscosity helped liquid CO_2_ reach the fabric’s interstices and remove contamination. The washing system was kept under high pressure, that helped in solubilizing the contaminants from the solvent at a low temperature. This made it a very suitable solvent for removing non-polar contamination.

## 5. Conclusions

The liquid CO_2_ wash was certainly effective in removing the targeted contaminants. The average washing efficiency for liquid CO_2_ was 95.36% which was significantly higher than the average washing efficiency of conventional wash: 68.77%. The controlled study included uniform contamination of the garments that helped understand and analyze both methods on the same level. One interesting trend that can be seen here when observing the conventional wash is that the cleaning efficiency and the $K_{OW}$ values have an inverse relation. This makes sense since the octanol-water partition coefficient indicates the ability of the compound to partition in the organic or aqueous phase. So, the higher value shows the reluctance of the compound to partition more towards the water. The results were statistically significant. A limitation of this study design is that the method was tested against liquid contamination and did not account for the particulate contamination that is experienced with smoke and soot in firefighter exposures. Additionally, studies have shown that the lack of mechanical action impedes the removal of particulate contamination for liquid CO_2_ [8]. Thus, it will be interesting to evaluate the efficacy of liquid CO_2_ when real-world samples are used. Simultaneously, it is important to investigate the redeposition of the contaminants while washing with this technique. Additionally, a further investigation of the impact of liquid CO_2_ on the durability of the turnout suit and its accessories is needed along with the operation costs for the method.

## Supplementary Material

Supplementary Materials Figure S1: Box-plot of average washing efficiencies of all compounds for all samples.
Figure S2: *t*-Test analysis (All samples).
Figure S3: Normality test when all samples are considered (Left: Conven-tional washing, Right: liquid CO_2_ washing)
Figure S4: Box-Plot of average washing efficiencies of all compounds (when equal number of samples are considered).
Figure S5: Normality test for equal number of samples (Left: Conventional washing, Right: liquid CO_2_ washing).
Figure S6: *t*-Test analysis (Equal number of samples)

## Figures and Tables

**Table 1.** Targeted Contaminants and their relevant properties.

Compound	Boiling Point (°C)	Volatile/Semi-volatile	Kow ^A^	LOD (ng/μL)	LOQ (ng/μL)	RSQ

Phenol	182	Volatile	1.46	0.29	0.90	0.9988
2,4,6-Trichlorophenol (2,4,6-TCP)	246	Volatile	3.69	0.17	0.52	0.9973
Pentachlorophenol (PCP)	310	Semi-volatile	5.12	0.22	0.67	0.9927
Di-butyl phthalate (DBP)	340	Semi-volatile	4.50	0.09	0.26	0.9998
Benzyl butyl phthalate (BBP)	370	Semi-volatile	4.73	0.10	0.30	0.9994
Di-ethyLhexyl phthalate (DEHP)	384	Semi-volatile	7.60	0.13	0.38	0.9997
Phenanthrene	340	Semi-volatile	4.46	0.22	0.67	0.9992
Pyrene	404	Semi-volatile	4.88	0.07	0.21	0.9997
Benzo[a] pyrene (BaP)	495	Semi-volatile	6.13	0.06	0.18	0.9995

A = values taken from PubChem^®^.

**Table 2.** Details of the liquid CO_2_ method.

Step	Details

Duration of cycle	50 min
Wash bath: Single wash	8 min
Rinse: Two cycles	4 min each
Pressure range	600–850 psi
Total load	50 lbs.
Detergent	Proprietary
CO2 grade	Beverage

**Table 3.** Ingredients of CD-1.

CD-1

D-Limonene
Non-ionic surfactant: 4-Nonylphenyl-polyethylene glycol
Mackamide C
Glycol ether

**Table 4.** Calibration Solutions for the chromatography method.

Calibration Standard	Target Concentration (ng/μL)	The Volume Injected from the Stock Solution (μL)	Mass per Unit Fabric Area (ng/cm^2^)

1	0.6	3	240
2	1.2	6	480
3	3	15	1200
4	6	18	2400
5	9	45	3600
6	12	60	4800

**Table 5.** Average washing efficiency of targeted contaminants for conventional and liquid CO_2_.

	Compounds	Conventional Wash	Count of ND ^a^	Liquid CO_2_	Count of ND ^a^

	Phenol	92.46% ^†^	5	92.46% ^†^	40
Phenols	2,4,6-tri-chlorophenol (TCP)	95.59% ^†^	5	95.59% ^†^	40
	Penta-chloro-phenol (PCP)	80.96%	0	94.43% ^†^	40
	Phenanthrene	92.32%	0	94.44% ^†^	40
PAHs	Pyrene	57.62%	0	98.22% ^†^	40
	Benzo[a]pyrene (BaP)	35.73%	0	98.52% ^†^	40
	Di-butyl-phthalate (DBP)	79.19%	0	97.80% ^†^	40
Phthalates	Benzyl-butyl-phthalate (BBP)	54.76%	0	97.08%	0
	Di-ethyl-hexyl-phthalate (DEHP)	30.29%	0	89.67%	0

† Non-detectable signal—$\frac{1}{2}$ LOQ used for calculation.

a = number of samples with non-detectable signals.

## Data Availability

All the data supporting the findings of the study are available within the article.
