# Supplementary material for: Comparative Analysis of the Liquid CO2 Washing with Conventional Wash on Firefighters’ Personal Protective Equipment (PPE)
Source: Textiles (Basel). Author manuscript; Available in PMC 2023 Dec 6. (PMC10698642; doi:10.3390/textiles2040036)
Supplement: Supplementary Materials — Figure S1: Box-plot of average washing efficiencies of all compounds for all samples. Figure S2: t-Test analysis (All samples). Figure S3: Normality test when all samples are considered (Left: Conven-tional washing, Right: liquid CO2 washing) Figure S4: Box-Plot of average washing efficiencies of all compounds (when equal number of samples are considered). Figure S5: Normality test for equal number of samples (Left: Conventional washing, Right: liquid CO2 washing). Figure S6: t-Test analysis (Equal number of samples) [file NIHMS1936382-supplement-Supplementary_Materials.pdf]

Liquid CO_2 All samples vs Conventional Wash

Average of All 40 samples for liquid CO_2 taken, average of all 5 samples for conventional wash

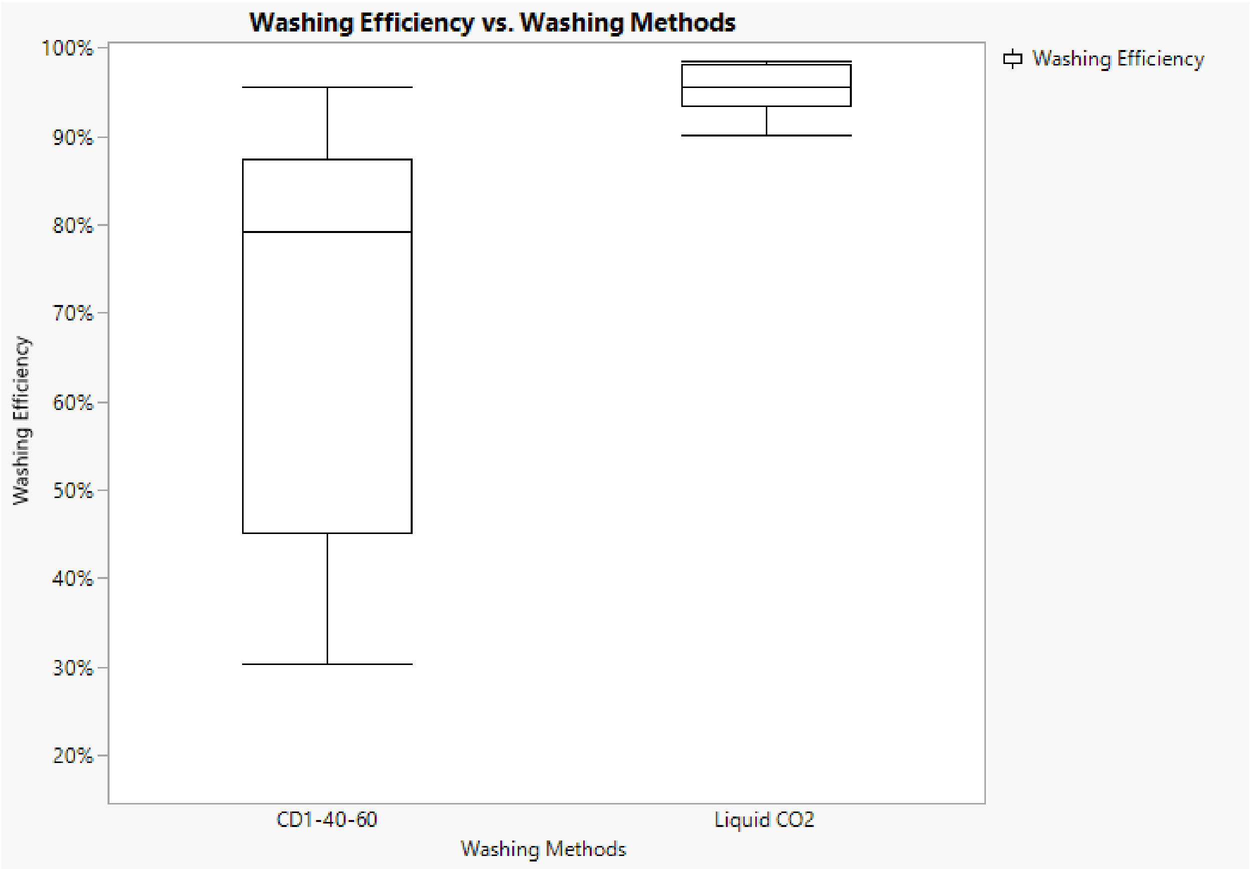

Figure S1: Box-plot of average washing efficiencies of all compounds for all samples

t Test

Liquid CO2-Conventional Wash

Assuming unequal variances

Difference	0.277433	t Ratio	3.44543
Std Err Dif	0.080522	DF	8.226654
Upper CL Dif	0.462231	Prob > |t|	0.0084*
Lower CL Dif	0.092636	Prob > t	0.0042*
Confidence	0.95	Prob < t	0.9958

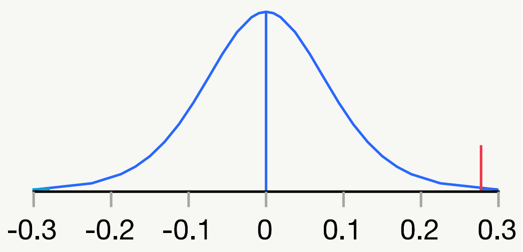

Figure S2: t-Test analysis (All samples)

Data Analysis

Conventional Wash

Goodness-of-Fit Test

	W	Prob<W
Shapiro-Wilk	0.9042853	0.2779

	A2	Simulated p-Value
Anderson-Darling	0.3968383	0.3076

Liquid CO_2

Goodness-of-Fit Test

	W	Prob<W
Shapiro-Wilk	0.9235319	0.4223

	A2	Simulated p-Value
Anderson-Darling	0.3036233	0.5416

Figure S3: Normality test when all samples are considered (Left: Conventional washing, Right: liquid CO2 washing)

Equal Number of Samples

5 samples randomly chosen from set of 40 samples for liquid CO_2, data for conventional wash is kept same

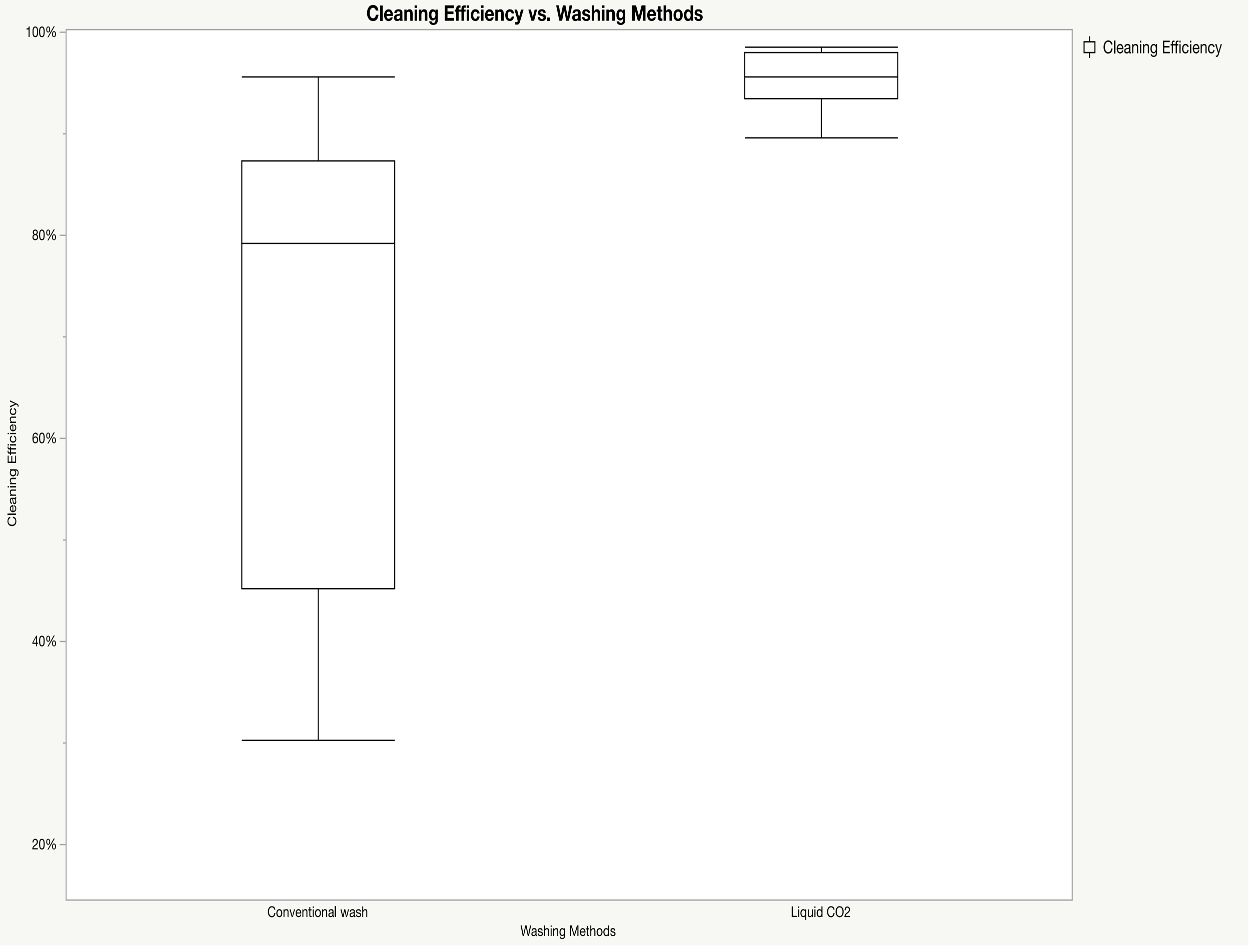

Figure S4: Box-Plot of average washing efficiencies of all compounds (when equal number of samples are considered)

Data Analysis

Conventional Wash

Goodness-of-Fit Test

	W	Prob<W
Shapiro-Wilk	0.9042853	0.2779

	A2	Simulated p-Value
Anderson-Darling	0.3968383	0.3144

Liquid CO_2

Goodness-of-Fit Test

	W	Prob<W
Shapiro-Wilk	0.9284053	0.4663

	A2	Simulated p-Value
Anderson-Darling	0.2811541	0.5988

Figure S5:Normality test for equal number of samples (Left: Conventional washing, Right: liquid CO_2 washing)

T-test

t Test

Liquid CO2-Conventional wash

Assuming unequal variances

Difference	0.276344	t Ratio	3.430912
Std Err Dif	0.080545	DF	8.236057
Upper CL Dif	0.461160	Prob > |t|	0.0086*
Lower CL Dif	0.091529	Prob > t	0.0043*
Confidence	0.95	Prob < t	0.9957

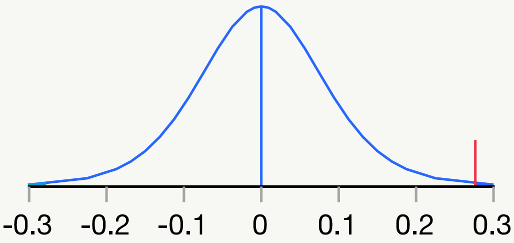

Figure S6:t-Test analysis (Equal number of samples)
